# Patterns of Ear Morbidities Among Pediatric Age Group: A Tertiary Care Hospital-Based Study

**DOI:** 10.7759/cureus.83958

**Published:** 2025-05-12

**Authors:** Shanila Feroz, Ejaz Ur Rehman, Muhammad Saad Raza, Mehrozia Nuzhat, Asma Noreen, Marion Samson

**Affiliations:** 1 ENT, United Medical and Dental College, Karachi, PAK; 2 Pediatrics, United Medical and Dental College, Karachi, PAK; 3 Pediatrics, Jinnah Medical and Dental College, Karachi, PAK

**Keywords:** karachi, otitis media, pattern, pediatric ear morbidities, public health, tertiary care hospital

## Abstract

Objective*:* To determine the prevalence of specific pediatric ear morbidities and assess their association with age and sex, while hypothesizing that there may be significant differences across demographic groups for pediatric ear morbidities.

Methodology*:* A cross-sectional study was conducted at Creek General Hospital and United Hospital, Karachi, from 2019 to 2023. A total of 788 children aged 4-12 years presenting with ear-related morbidities were enrolled using consecutive sampling. Data were collected using a structured evaluation form that captured demographic information and diagnostic categories. Statistical analyses were performed with SPSS version 27, utilizing descriptive statistics and Fisher’s exact test to examine associations (p ≤ 0.05).

Results*:* Among the 788 participants, 49.24% (388) were male and 50.76% (400) female, with a majority aged 8-12 years. The most frequent diagnosis was otitis media with effusion (OME) 41.24% (325), followed by chronic suppurative otitis media (CSOM) 19.29% (152) and acute otitis media (AOM) 15.48% (122). Less common conditions included otitis externa 9.26% (73), impacted cerumen 10.41% (82), and foreign body presence in the ear 2.54% (20). Overall, ear morbidities showed no significant variation by age or sex, except for impacted earwax and AOM, which exhibited age- and sex-related differences, respectively (P-value <0.05).

Conclusion*:* OME, CSOM, and AOM emerged as the most prevalent ear morbidities among pediatric patients in tertiary care settings. These findings suggest that early diagnosis and timely intervention may help mitigate complications and are therefore recommended as important public health measures. Efforts to address underlying factors such as inadequate healthcare access and poor hygiene in socioeconomically disadvantaged areas are crucial. Further research focusing on regional disparities and preventive strategies is recommended.

## Introduction

Ear morbidities are a significant public health concern, particularly in the pediatric population, where they can have a profound impact on communication, education, and overall quality of life [1]. These conditions range from common infections such as otitis media to congenital anomalies and chronic conditions like hearing loss, each presenting unique challenges in diagnosis and management. Globally, ear morbidities contribute substantially to the burden of disease in children, often leading to developmental delays if left untreated [2-3]. Tertiary care hospitals play a critical role in managing pediatric ear morbidities, serving as referral centers for specialized diagnosis and treatment [4]. Understanding the patterns of ear morbidities among children in these settings is essential for developing targeted interventions and allocating healthcare resources effectively [5-6]. In Pakistan, pediatric ear morbidities are a growing concern, given the high prevalence of risk factors such as poor hygiene, lack of awareness, delayed access to healthcare, and socioeconomic disparities [7-8]. Limited availability of specialized healthcare facilities, especially in rural and underprivileged areas, further exacerbates the issue. According to various studies, conditions such as otitis media are among the leading causes of hearing impairment in Pakistani children, often resulting in preventable complications due to late diagnosis and inadequate treatment [9-10]. Despite the prevalence of ear-related disorders in children, there remains a gap in region-specific data, particularly in developing countries, such as Pakistan, where access to healthcare services may be limited. Therefore, the objective of this study was to determine the prevalence of various ear morbidities among pediatric patients in tertiary care hospitals of Karachi, and to identify any age- or sex-related pattern/association in their occurrence, hypothesizing that there may be significant demographic differences in their occurrence.

## Materials and methods

This cross-sectional study was carried out at Creek General Hospital and United Hospital, Karachi, Pakistan, over a 5-year period from 2019 to 2023. Ethical approval was granted by the Institutional Review Board of United Medical and Dental College (UMDC/Ethics 254), ensuring compliance with the principles outlined in the Declaration of Helsinki. A minimum sample size of 384 participants were calculated using the OpenEpi infinite sample size calculator, with a confidence level of 95% and a margin of error of 5%, but a total of 788 pediatric participants were enrolled using consecutive sampling, to include all the participants that fits the criteria within the duration of study data. OpenEpi was specifically chosen due to its wide acceptance in epidemiological research, user-friendly interface, and validated algorithms that offer reliable sample size estimates, particularly suited for large or undefined populations such as the pediatric cohort in this study. Inclusion criteria comprised children aged 4-12 years presenting with ear morbidities at the tertiary care hospitals, whose parents or guardians provided informed consent. Exclusion criteria included individuals with a history of ear surgeries, incomplete medical records, or those who declined participation. A pilot study with 20 participants was conducted to evaluate the feasibility and reliability of the data collection instrument, though the pilot data were excluded from the final analysis. Data collection was facilitated by a structured evaluation form specifically developed for this study (Appendices: Table 3). The form documented demographic variables (age, sex), diagnostic categories (e.g., chronic suppurative otitis media (CSOM), otitis media with effusion (OME), acute otitis media (AOM), otitis externa, impacted earwax, foreign bodies, or other conditions), and allowed for additional observations. Participants were recruited during routine visits to the outpatient department (OPD). The diagnostic criteria for each ear morbidity were based on standard otolaryngological guidelines and clinical assessment protocols. For instance, diagnoses of OME, foreign body in ear, impacted earwax, otitis externa, and AOM were confirmed based on otoscopic findings such as the presence of effusion, erythema, and tympanic membrane bulging, while CSOM was diagnosed based on persistent otorrhea through a perforated tympanic membrane for more than six weeks. Prior to data collection, informed consent was obtained from parents or guardians. Confidentiality was rigorously maintained, with all participant data anonymized and accessible exclusively to authorized researchers. Data were manually entered into Microsoft Excel and analyzed using SPSS version 27. Descriptive statistics summarized categorical variables as frequencies and percentages, while continuous variables were reported as means and standard deviations. Relationships between variables were examined using Fisher’s exact test, with statistical significance set at p < 0.05. The study adhered to stringent ethical protocols to ensure participant privacy and followed Strengthening the Reporting of Observational studies in Epidemiology (STROBE) guidelines for comprehensive and standardized reporting.

Operational definitions

Otitis Media with Effusion (OME)

Presence of fluid in the middle ear without signs or symptoms of acute ear infection, confirmed via otoscopic examination showing a dull or retracted tympanic membrane with air-fluid levels or bubbles, persisting for more than 3 weeks.

Acute Otitis Media (AOM)

Acute onset of ear pain (otalgia), erythema, and bulging of the tympanic membrane observed on otoscopy, often accompanied by fever or recent upper respiratory tract infection. Symptom duration was typically less than 3 weeks.

Chronic Suppurative Otitis Media (CSOM)

Persistent otorrhea through a perforated tympanic membrane lasting for 6 weeks or longer, confirmed by otoscopic findings.

Otitis Externa

Inflammation of the external auditory canal is characterized by ear pain, tenderness, and erythema, typically with a symptom duration of less than 3 weeks.

Impacted Cerumen

Accumulation of earwax causes obstruction of the ear canal and leading to symptoms such as hearing loss or ear fullness, diagnosed through direct visualization on otoscopy.

Foreign Body in Ear

Presence of any non-biological object in the external auditory canal, confirmed on otoscopic examination.

## Results

Of the 788 participants, 49.24% were male, and 50.76% were female. The majority of the participants were between 8-12 years of age, with the remaining falling into the 4-7 years age group (Table 1).

**Table 1:** Demographic data of the participants.

Variables	Frequency (n)	Percentage (%)
Gender (N=788)		
Male	388	49.24
Female	400	50.76
Age (N=788)		
4-7 years	389	49.37
8-12 years	399	50.63

**Figure 1:**
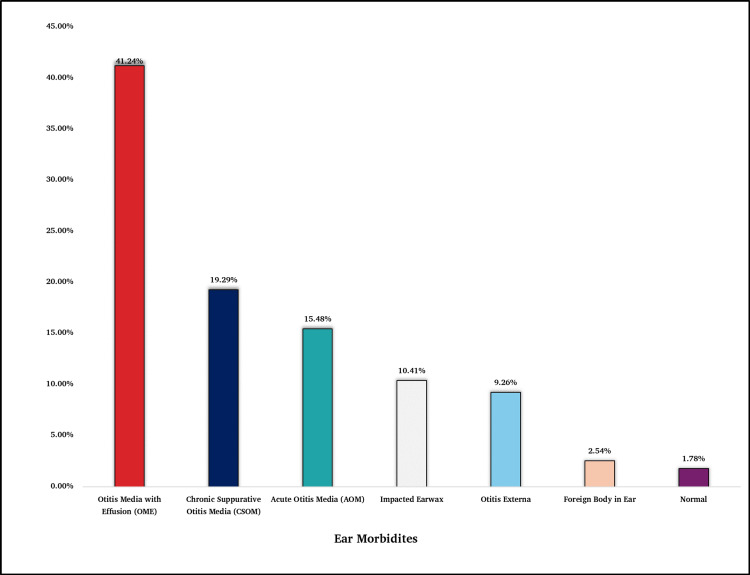
Prevalence of Pediatric Ear Morbidities (N=788)

The most prevalent condition among participants was OME 41.24% (325), followed by chronic suppurative otitis media (CSOM) 19.29% (152) and acute otitis media (AOM) 15.48% (122). A smaller subset of participants presented with otitis externa 9.26% (73), impacted cerumen 10.41% (82), and foreign body presence in the ear 2.54% (20) (Figure 1).

This trend was further observed across pediatric age groups and sex. While most ear morbidities did not show statistically significant differences between age groups or sexes, notable exceptions were observed. Impacted earwax was significantly more frequent among children aged 8-12 years (p=0.008), and males exhibited a significantly higher prevalence of AOM compared to females (p=0.011). For other conditions, including OME, CSOM, otitis externa, and foreign bodies, no significant difference with age or sex was identified (Table 2).

**Table 2:** Association of pediatric age group and sex with ear comorbidities (N=788) Note: Fisher’s exact test is applied to show the statistical difference. A P-value of <0.05 is considered significant.

Variables	Age Group n (%)		P-value	Sex n (%)		P-value
	4-7 years	8-12 years		Male	Female	
Ear Morbidities						
Acute otitis media (AOM)	60 (15.4%)	62 (15.5%)	0.969	73 (18.81%)	49 (12.25%)	0.011
Chronic suppurative otitis media (CSOM)	68 (17.5%)	84 (21.1%)	0.201	73 (18.81%)	79 (19.75%)	0.738
Foreign body in ear	11 (2.8%)	9 (2.3%)	0.656	11 (2.84%)	9 (2.25%)	0.599
Impacted earwax	29 (7.5%)	53 (13.3%)	0.008	37 (9.54%)	45 (11.25%)	0.432
Normal	6 (1.5%)	8 (2.0%)	0.593	6 (1.55%)	8 (2.00%)	0.633
Otitis externa	42 (10.8%)	31 (7.8%)	0.147	34 (8.76%)	39 (9.75%)	0.632
Otitis media with effusion (OME)	173 (44.5%)	152 (38.1%)	0.068	154 (39.69%)	171 (42.75%)	0.383

## Discussion

This study explored the patterns of ear morbidities in pediatric patients attending tertiary care hospitals in Karachi, Pakistan, over a five-year period. Our findings provide valuable insights into the prevalence and demographic trends of ear conditions such as OME, CSOM, and AOM in children, emphasizing the importance of early diagnosis and tailored intervention strategies.

Among the conditions identified, OME emerged as the most prevalent diagnosis, affecting 44.5% of children aged 4-12 years. This high prevalence aligns with global [11-12] and local studies [7,13], indicating that OME is a leading cause of hearing loss and communication challenges in children, particularly in low- and middle-income countries like Pakistan, where access to healthcare is often delayed. This prevalence can be attributed to factors such as delayed healthcare-seeking behavior, poor sanitation, and higher rates of respiratory infections common in low- and middle-income settings [14-17]. Additionally, the lack of significant differences in the prevalence of OME across age groups or sexes further emphasizes its widespread impact in this population. These findings highlight the critical need for early screening programs to detect and manage OME before it progresses to complications such as speech delays or learning difficulties.

Furthermore, CSOM was the second most common diagnosis, accounting for 19.3% of cases. This aligns with research indicating that CSOM is a significant contributor to hearing loss in children worldwide as well as locally, particularly in regions with inadequate sanitation and healthcare access [18-20]. The persistence of CSOM in this cohort may be attributed to delays in diagnosis and treatment, compounded by environmental factors such as high humidity and poor hygiene conditions in Karachi [21-23]. Addressing these challenges through public health campaigns focused on improving hygiene and increasing awareness about the importance of timely medical intervention is essential.

Moreover, AOM accounted for 15.7% of ear morbidities, which is consistent with findings from other local studies [24-25]. While AOM is often self-limiting, recurrent episodes can predispose children to chronic conditions such as OME or CSOM [26]. This underscores the importance of preventive strategies, including vaccination programs and public education about the risks associated with untreated ear infections.

Additionally, less common conditions, including otitis externa (9.8%), impacted earwax (10.4%), and foreign body presence (2.5%), were also identified. The prevalence of impacted earwax was significantly higher among children aged 8-12 years compared to younger children, which may reflect differences in behavioral or anatomical factors [27]. These findings emphasize the importance of regular ear examinations in pediatric populations to identify and manage such conditions promptly.

The lack of statistically significant associations between gender and most ear morbidities suggests that these conditions affect boys and girls relatively equally in this setting, in congruence with literature [28] However, the slight predominance of some conditions in females, such as OME, may be linked to biological and hormonal differences, as well as sociocultural factors influencing healthcare-seeking behaviors for girls [29], which warrants further investigation to understand potential gender-specific factors.

Our study's findings underscore the critical role of tertiary care hospitals in managing pediatric ear morbidities in urban centers like Karachi. The high prevalence of preventable conditions such as OME and CSOM highlights the need for improved access to early diagnosis and specialized care. Additionally, targeted public health interventions addressing risk factors such as poor hygiene and limited healthcare access in socioeconomically disadvantaged areas could significantly reduce the burden of ear morbidities in this population.

This study has several limitations. The use of consecutive sampling may not fully represent the broader population dynamics of ear morbidities in Karachi. Furthermore, the focus on urban tertiary care hospitals limits the generalizability of our findings to rural or underprivileged populations, where access to healthcare is more restricted. Future studies should aim to include a more diverse geographic scope and utilize random sampling methods to enhance representativeness. Additionally, the exclusion of children with a history of ear surgeries or ongoing antibiotic treatment may have skewed the observed prevalence rates of certain conditions.

## Conclusions

In conclusion, our study provides valuable insights into the patterns of pediatric ear morbidities in tertiary care settings in Karachi. Based on the high prevalence identified, early diagnosis and timely intervention are recommended public health measures to potentially mitigate complications associated with conditions such as OME and CSOM. Addressing these challenges through evidence-based strategies can improve outcomes for children and reduce the burden of ear morbidities in low- and middle-income countries.

## Appendices

**Table 3:** Evaluation form

Section 1: Demographic Information
Participant ID:
Do you consent to participate in this study?
Yes
No
Age:
11-20 years
21-30 years
31-40 years
41-50 years
51-60 years
61 years and above
Sex:
Male
Female
Other:_______________________
Occupation: _______________________
Residence:
Urban
Rural
Section 2: Diagnosis
Diagnosis:
Chronic suppurative otitis media (CSOM)
Otitis Externa
Both
Section 3: Pathogen Characterization
Pathogen type (Select one):
Bacterial
Fungal
Both
If a bacterial pathogen is identified, specify:
*Staphylococcus aureus*
*Pseudomonas aeruginosa*
*Streptococcus pneumoniae*
*Escherichia coli*
Other: ______________________
If a fungal pathogen is identified, specify:
*Candida spp.*
*Aspergillus spp.*
*Fusarium spp.*
Other: ______________________

## References

[1] Gupta PC, Chakravarty S (2019). An epidemiological study on the morbidities of ear among children near a rural practicing field area of a tertiary medical institute. Asian Pac J Health Sci.

[2] Bhatia R, Chauhan A, Kaur K, Rana M, Pradhan P, Singh M (2022). Prevalence of ear infections in children (0 to 15 years) of India: A systematic review and meta-analysis. Int Arch Public Health Community Med.

[3] Jin Y, Yang X, Sun H (2024). Global, regional, and national burdens of otitis media from 1990 to 2019: A population based study. Ear Hear.

[4] Puricelli MD (2024). Comprehensive management of chronic ear disease: Consecutive patient analysis at a tertiary children's hospital. Int J Pediatr Otorhinolaryngol.

[5] Shekhar H, Khokhar A, Motwani G, Daral S (2022). Prevalence of ear morbidities among school children in Delhi, India: A cross-sectional study. Int J Adolesc Med Health.

[6] Maharjan M, Phuyal S, Shrestha M, Bajracharya R (2020). Chronic otitis media and subsequent hearing loss in children from the Himalayan region residing in Buddhist Monastic schools of Nepal. J Otol.

[7] Riaz N, Ajmal M, Khan MS (2022). Frequency of otitis media with effusion among children aged 1-5 years presenting to immunization center of tertiary care hospitals, Rawalpindi. World J Otorhinolaryngol Head Neck Surg.

[8] Bhatti MA, Shaikh SA, Memon SF, Aamir K, Ramzan A, Shah H (2024). Frequency of hearing impairment in school-going children of district Hyderabad, Sindh, Pakistan: Frequency of hearing impairment. Pak J Health Sci.

[9] Ali S, Khan MS, Zahid L (2022). Common causes of hearing impairment among children less than 8 years of age in District Bahawalpur, Punjab Pakistan. J Pak Med Assoc.

[10] Babar A, Fatima M, Fatima Z, Batool S, Khan MS, Mukhtar F, Mazhar F (2023). Prevalence of hearing impairment with respect of age and gender in patient with otitis media. J Health Rehab Res.

[11] Restuti RD, Tamin S, Nugroho DA, Hutauruk SM, Mansyur M (2022). Factors affecting the occurrence of otitis media with effusion in preschool and elementary school children: A comparative cross-sectional study. BMJ Open.

[12] Jamal A, Alsabea A, Tarakmeh M (2022). Effect of ear infections on hearing ability: A narrative review on the complications of otitis media. Cureus.

[13] Anwar K, Mohammad AY, Khan S (2023). Spontaneous resolution of ‘glue ear’in children-An experience at a tertiary care teaching hospital of Khyber Pakhtunkhwa: Spontaneous resolution of glue ear in children. Pak J Health Sci.

[14] Mahmood H, Khan SM, Saleem Abbasi YS (2017). Healthcare seeking trends in acute respiratory infections among children of Pakistan. World J Clin Infect Dis.

[15] Huda M, Rabbani F, Shipton L, Aftab W, Khan KS, Marini MG (2023). Listening to caregivers: narratives of health seeking for children under five with pneumonia and diarrhea: insights from the NIGRAAN trial in Pakistan. J Multidiscip Healthc.

[16] Shaikh BT, Hatcher J (2007). Health seeking behaviour and health services utilization trends in national health survey of Pakistan: What needs to be done?. J Pak Med Assoc.

[17] Hussain R, Rashidian A, Hafeez A, Mirzaee N (2019). Factors influencing healthcare seeking behaviour at primary healthcare level, in Pakistan. J Ayub Med Coll Abbottabad.

[18] Hussain A, No S (2009). Prevalence and comparison of chronic suppurative otitis media in government and private schools. Ann Pak Inst Med Sci.

[19] Adhikari P, Joshi S, Baral D, Kharel B (2009). Chronic suppurative otitis media in urban private school children of Nepal. Braz J Otorhinolaryngol.

[20] Hunt L, Mulwafu W, Knott V (2017). Prevalence of paediatric chronic suppurative otitis media and hearing impairment in rural Malawi: A cross-sectional survey. PLoS One.

[21] Haqdad M, Aftab AA, Kumar A, Ali SS, Rai D, Ahmed N (2022). Evaluation of complications and management of chronic suppurative otitis media: A retrospective study. Pak J Med Health Sci.

[22] Ghani S, Qasim Z, Mughal T, Haq E, Husain S (2015). Bacteriological prevalence and growth pattern in patients of chronic suppurative otitis media in Mirpur AJK. JIMDC.

[23] Morris P (2012). Chronic suppurative otitis media. BMJ Clin Evid.

[24] Ahmed I, Lakhiar AA, Yousuf S, Kumar A, Ashraf MA, Chaudhry AR (2022). Risk factors of acute otitis media in early age (A Meta-Analysis). Pak J Med Health Sci.

[25] Raza MS, Sangi R, Hanif S, Haque T, Jamro S, Memon MH (2022). Prevalence of acute otitis media in febrile children at a tertiary care hospital. Pak J Med Health Sci.

[26] Danishyar A, Ashurst JV (2025). Acute Otitis Media. https://www.ncbi.nlm.nih.gov/books/NBK470332/.

[27] Tolan MM, Choi JS, Tibesar MT, Adams ME (2024). Cerumen impaction: Prevalence and associated factors in the United States population. Laryngoscope Investig Otolaryngol.

[28] Gupta K, Goyal GK, Garg S (2020). An epidemiological study of ear morbidities among primary school children in a rural area of Delhi. Int J Health Clin Res.

[29] Enoksson F, Ruiz Rodriguez A, Peno C (2020). Niche- and gender-dependent immune reactions in relation to the microbiota profile in pediatric patients with otitis media with effusion. Infect Immun.

